# Does epilepsy always indicate worse outcomes? A longitudinal follow-up analysis of 485 glioma patients

**DOI:** 10.1186/s12957-022-02772-2

**Published:** 2022-09-19

**Authors:** Honglin Ge, Guangfu Di, Zheng Yan, Dongming Liu, Yong Liu, Kun Song, Kun Yang, Xinhua Hu, Zijuan Jiang, Xiao Hu, Lei Tian, Chaoyong Xiao, Yuanjie Zou, Hongyi Liu, Jiu Chen

**Affiliations:** 1 Department of Neurosurgery, the Affiliated Brain Hospital of Nanjing Medical University, Nanjing, 210029 Jiangsu China; 2 Department of Neurosurgery, the First Affiliated Hospital (Yijishan Hospital), Wannan Medical College, Wuhu, China; 3 Department of Pathology, the Affiliated Brain Hospital of Nanjing Medical University, Nanjing, 210029 Jiangsu China; 4 Institute of Brain Functional Imaging, Nanjing Medical University, Nanjing, 210029 Jiangsu China; 5 Department of Radiology, the Affiliated Brain Hospital of Nanjing Medical University, Nanjing, 210029 Jiangsu China; 6 Institute of Neuropsychiatry, the Affiliated Brain Hospital of Nanjing Medical University, Fourth Clinical College of Nanjing Medical University, Nanjing, 210029 Jiangsu China

**Keywords:** Brain tumor, Perioperative outcomes, Glioma, Survival, Epilepsy

## Abstract

**Background:**

Epilepsy is one of the most common glioma complications, and the two may be connected in more ways than we understand. We aimed to investigate the clinical features of glioma-associated epilepsy and explore the risk factors associated with it.

**Methods:**

We collected clinical information from 485 glioma patients in the Nanjing Brain Hospital and conducted 4 periodic follow-up visits. Based on the collected data, we analyzed the clinical characteristics of glioma patients with or without epilepsy and their relationship with survival.

**Results:**

Among glioma patients, younger people were more likely to have epilepsy. However, epilepsy incidence was independent of gender. Patients with grade II gliomas were most likely to develop epilepsy, while those with grade IV gliomas were least likely. There was no difference in Karnofsky Performance Status scores between patients with glioma-associated epilepsy and those without epilepsy. Additionally, epilepsy was independently associated with longer survival in the World Health Organization grade IV glioma patients. For grades II, III, and IV tumors, the 1-year survival rate of the epilepsy group was higher than that of the non-epilepsy group.

**Conclusions:**

Epilepsy did not lead to worse admission performance and correlated with a better prognosis for patients with grade IV glioma.

**Supplementary Information:**

The online version contains supplementary material available at 10.1186/s12957-022-02772-2.

## Introduction

Glioma is one of the most common neurosurgical diseases, accounting for about a quarter of the primary brain tumors [1]. Gliomas place a massive burden on the patients’ physical, psychological, and financial statuses [2–4]. Epilepsy is a potential glioma complication [5], occurring in up to 60–85% of low-grade gliomas (LGGs) [6]. Patients with glioblastoma (GBM), the most aggressive form of glioma [1], also have a high incidence of epilepsy (30–60%) [6].

Epilepsy adversely affects patients’ quality of life and neurocognitive functions [7]. It can present in the form of common convulsions or unusual symptoms such as auditory hallucinations and phantom smells [8]. People with epilepsy have a higher mortality risk than the general population [9]. Epilepsy in patients with glioma may result in hospitalization or may be detected during post-admission examinations.

Both glioma and epilepsy have underlying mechanisms that are beyond our current understanding. Epilepsy occurs at a high rate in glioma patients but not in patients with all types of brain tumors. The incidence of epilepsy in patients with meningiomas, the most common brain tumor, is only 26–31% [10]. Gliomas have been known to induce epilepsy in various ways, such as by releasing glutamate [11–13]. However, recently, epilepsy was found to promote glioma growth [14]. In other words, epilepsy and glioma are more closely linked than usually realized.

The relationship between glioma and epilepsy is not fully understood yet. To better understand glioma-related epilepsy, we analyzed the general characteristics of tumor patients with and without epilepsy and focused on the relationship between epilepsy and glioma prognosis.

## Methods

### Patient identification

We enrolled 485 glioma patients (confirmed by histopathology) from January 2015 to December 2020. Patients with confirmed neuronal and mixed neuronal-glial tumors and who had only received biopsies were excluded. Four-year follow-ups were conducted in May 2017, October 2019, April 2020, and December 2021. During the follow-ups, 244 patients died, while the remaining 241 are still alive. As this study was retrospective, informed consent was not obtained.

### Demographics, clinical assessments, and tumor features

We collected general information of patients, including age, sex, onset symptoms, and Karnofsky Performance Status (KPS) scores at the time of their admission and discharge. A KPS score of < 70 was defined as poor performance. The onset symptoms included focal deficits, epilepsy, cognitive changes, and headaches. Focal deficits referred to motor dysfunction, somatosensory impairment, and cranial nerve dysfunction. Cognitive change involved a decline in cognitive ability or changes in personality or behavior. Notably, epilepsy included pre-admission convulsions and some other behaviors or symptoms recognized as epileptic by an electroencephalogram (EEG) after admission and before surgery. Thus, we were referring to preoperative epilepsy, not postoperative. Imaging reports of the brain, including MRI and CT scans, were independently reviewed by radiologists with more than 5 years of experience to determine the location and number of tumors. We recorded the lobe in which each glioma was located. If the glioma spread across more than one lobe, we recorded in which lobe it was primarily located. When gliomas were not located in any lobe, we recorded them as “others.” Simultaneously, we reviewed reports by senior pathologists to assess the pathological type and the World Health Organization (WHO) grade of the gliomas. The highest grade was recorded when patients had multiple tumors with different grades or different grades within a single tumor.

### Statistical analyses

All statistical analyses were performed using R software (4.1.0). The data were reported as mean ± standard deviation (SD) for continuous variables and percentages for categorical variables. The chi-squared test and Fisher’s exact test were utilized for comparing categorical variables between groups. Nonparametric tests were used to assess the differences among continuous variables. Logistic regression was used to analyze the risk factors of epilepsy and calculate the odds ratios (OR) and 95% confidence intervals (CI). The Kaplan–Meier (KM) method was used to calculate the 1-year survival rate of patients in each group. Cox proportional risk models were used to determine the effects of variables on glioma survival and calculate hazard ratios (HR) and 95% CI. Both logistic regression and Cox risk regression need univariate and multivariate analysis. Due to the many variables, we pre-screened them by performing a univariate analysis. In pre-screening, the threshold can be appropriately relaxed [15], and variables with a *p*-value of < 0.1 in univariate analysis were entered into the multivariate analysis. In the multivariate model, *p* < 0.05 was considered statistically significant. In the multivariate model, an OR or HR of < 1 indicated that the variable was a protective factor of the dependent variable. An OR or HR of > 1 indicated that the variable was a risk factor of the dependent variable.

## Result

### Demographics, clinical assessments, and tumor features

In terms of anatomical locations, epilepsy incidence was higher in patients with supratentorial tumors but rare in those with posterior fossa tumors. Recurrent tumors or multiple tumors did not affect epilepsy incidence. Similarly, epilepsy patients did not show worse admission performance status. Epilepsy was significantly associated with WHO grade II glioma tumors in a multivariate model. Patients with grade I and III tumors also had a higher epilepsy incidence than those with grade IV tumors (Table 1).

**Table 1:** General characteristics

Variables	Groups	Epilepsy	No epilepsy	Total	Univariate *p*-value	Multivariate *p*-value	Multivariate OR (95% *CI*)
**Gender**	**Female**	**59 (12.16%)**	**181 (37.32%)**	**240 (49.48%)**	**Reference**	**-**	**-**
	**Male**	**60 (12.37%)**	**185 (38.14%)**	**245 (50.52%)**	**0.981**	**-**	**-**
**Age (years)**		**43.96 ± 16.98**	**54.28 ± 15.07**	**51.75 ± 16.18**	**0.000**	**0.000**	**0.99 (0.98–0.99)**
**Location**	**Supratentorial**	**117 (24.12%)**	**332 (68.45%)**	**449 (92.58%)**	**0.006**	**0.000**	**1.36 (1.18–1.57)**
	**Subtentorial**	**2 (0.41%)**	**34 (7.01%)**	**36 (7.42%)**	**Reference**	**Reference**	**Reference**
**Tumor number**	**Single**	**110 (22.68%)**	**331 (68.25%)**	**441 (90.93%)**	**Reference**	**-**	**-**
	**Multiple**	**9 (1.86%)**	**35 (7.22%)**	**44 (9.07%)**	**0.510**	**-**	**-**
**Admission status**	**KPS ≤ 70**	**15 (3.09%)**	**70 (14.43%)**	**85 (17.53%)**	**0.105**	**-**	**-**
	**KPS > 70**	**104 (21.44%)**	**296 (61.03%)**	**400 (82.47%)**	**Reference**	**-**	**-**
**Type**	**Primary**	**106 (21.86%)**	**320 (65.98%)**	**426 (87.84%)**	**Reference**	**-**	**-**
	**Recurrent**	**13 (2.68%)**	**46 (9.48%)**	**59 (12.16%)**	**0.634**	**-**	**-**
**WHO grade**	**I**	**12 (2.47%)**	**28 (5.77%)**	**40 (8.25%)**	**0.015**	**0.079**	**1.14 (0.98–1.33)**
	**II**	**55 (11.34%)**	**53 (10.93%)**	**108 (22.27%)**	**0.000**	**0.000**	**1.37 (1.24–1.50)**
	**III**	**19 (3.92%)**	**68 (14.02%)**	**87 (17.94%)**	**0.087**	**0.072**	**1.10 (0.99–1.21)**
	**IV**	**33 (6.80%)**	**217 (44.74%)**	**250 (51.55%)**	**Reference**	**Reference**	**Reference**
**Total**		**119 (24.54%)**	**366 (75.46%)**	**485**			

Among grades I and II, patients with temporal gliomas were the most prone to have epilepsy. In grade III, patients with insular glioma were the most likely to have epilepsy, followed by those with temporal glioma. Young people with WHO grades I and II gliomas were more likely to develop epilepsy. Men with WHO grade III glioma were more likely to develop epilepsy. There were no variables associated with epilepsy in patients with grade IV glioma (Supplementary Tables 1, 2, 3 and 4). Compared to LGGs, high-grade gliomas are more likely to occur in the brain lobes (except the insula) (Supplementary Table 5).

### Symptoms of glioma patients

**Fig. 1:**
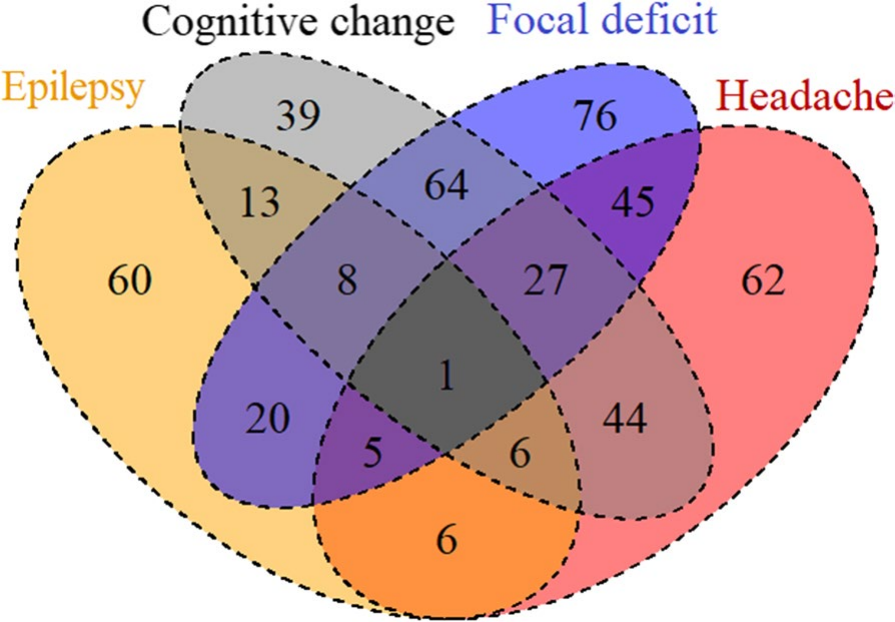
Venn diagram of symptoms in glioma patients. The figure shows the distribution of different symptoms among the patients

Focal deficits were the most common symptom reported in 246 cases out of a total of 485, followed by cognitive changes (202 cases), headache (196 cases), and epilepsy (119 cases) (Fig. 1).

The most frequent combination of symptoms was focal deficits combined with cognitive changes, accounting for 100 out of 485. Only one patient presented all four symptoms simultaneously. Epileptic symptoms in glioma patients tended to appear alone. Sixty of 119 patients with epilepsy had no other symptoms, accounting for 12.37% of all glioma patients. The most common concomitant symptom of epilepsy was cognitive changes, and the least common was a headache.

### Tumor characteristics

Among the 485 patients, diffuse astrocytic and oligodendroglial tumors (including various oligodendrogliomas, diffuse astrocytomas, and GBMs) were reported in 418 cases and were classified as grades II, III, and IV tumors. Other astrocytic and ependymal tumors were reported in 49 and 18 cases, respectively, and classified as WHO grades I, II, and III tumors.

In more detailed pathological classifications, GBMs were the most common type, reported in 250 cases. The most common subtype of GBM was isocitrate dehydrogenase (IDH) wild-type GBM, reported in 157 cases, whereas IDH-mutant GBM was reported in only 15 cases.

Age was associated with a higher grade of tumors. There was no significant difference in gender composition among the different grades (Table 2).

**Table 2:** Pathological type

WHO grade	Histologic type	Age	Gender (female/male)	M-code	No	% in grade	% in total
**I**	**Pilocytic astrocytoma**			**M9421/1**	**37**	**92.50%**	**7.63%**
	**Subependymoma**			**M9383/1**	**3**	**7.50%**	**0.62%**
	**Total**	**33.93 ± 17.31**	**21/19**		**40**		**8.25%**
**II**	**Diffuse astrocytoma (IDH mutant)**			**M9400/3**	**17**	**15.74%**	**3.51%**
	**Diffuse astrocytoma (IDH wild type)**			**M9400/3**	**15**	**13.89%**	**3.09%**
	**Diffuse astrocytoma (NOS)**			**M9400/3**	**15**	**13.89%**	**3.09%**
	**Oligodendroglioma (IDH mutant and 1p/19q-codeleted)**			**M9450/3**	**29**	**26.85%**	**5.98%**
	**Oligodendroglioma (NOS)**			**M9450/3**	**7**	**6.48%**	**1.44%**
	**Oligoastrocytoma (NOS)**			**M9382/3**	**11**	**10.19%**	**2.27%**
	**Pilomyxoid astrocytoma**			**M9425/3**	**2**	**1.85%**	**0.41%**
	**Pleomorphic xanthoastrocytoma**			**M9424/3**	**8**	**7.41%**	**1.65%**
	**Ependymoma**			**M9391/3**	**4**	**3.70%**	**0.82%**
	**Total**	**43.48 ± 16.28**	**64/44**		**108**		**22.26%**
**III**	**Anaplastic astrocytoma (IDH mutant)**			**M9401/3**	**10**	**11.49%**	**2.06%**
	**Anaplastic astrocytoma (IDH wild type)**			**M9401/3**	**20**	**22.99%**	**4.12%**
	**Anaplastic astrocytoma (NOS)**			**M9401/3**	**7**	**8.05%**	**1.44%**
	**Anaplastic oligodendroglioma (IDH mutant and 1p/19q-codeleted)**			**M9451/3**	**13**	**14.94%**	**2.68%**
	**Anaplastic oligodendroglioma (NOS)**			**M9451/3**	**7**	**8.05%**	**1.44%**
	**Anaplastic oligoastrocytoma (NOS)**			**M9382/3**	**17**	**19.54%**	**3.51%**
	**Anaplastic pleomorphic xanthoastrocytoma**			**M9424/3**	**2**	**2.30%**	**0.41%**
	**Anaplastic ependymoma**			**M9392/3**	**11**	**12.64%**	**2.27%**
	**Total**	**51.39 ± 15.58**	**40/47**		**87**		**17.93%**
**IV**	**GBM (IDH wild type)**			**M9440/3**	**157**	**62.80%**	**32.37%**
	**Gliosarcoma**			**M9442/3**	**5**	**2.00%**	**1.03%**
	**Epithelioid GBM**			**M9443/3**	**1**	**0.40%**	**0.21%**
	**GBM (IDH mutant)**			**M9440/3**	**15**	**6.00%**	**3.09%**
	**GBM (NOS)**			**M9440/3**	**72**	**28.80%**	**14.85%**
	**Total**	**58.30 ± 11.74**	**115/135**		**250**		**51.55%**
**Total**		**51.75 ± 16.18**	**240/245**		**485**		

### Association between epilepsy and survival

The Cox risk regression model was used to study the effects of epilepsy on glioma patient survival. To eliminate the influence of possible confounding factors on survival analysis, we included basic variables in the model. To be clear, only one patient died out of 40 grade I glioma patients. A survival analysis of this data was unreasonable, so we excluded grade I tumors. As the WHO grade is highly correlated with tumor survival [1], different grades of gliomas were analyzed separately.

**Fig. 2:**
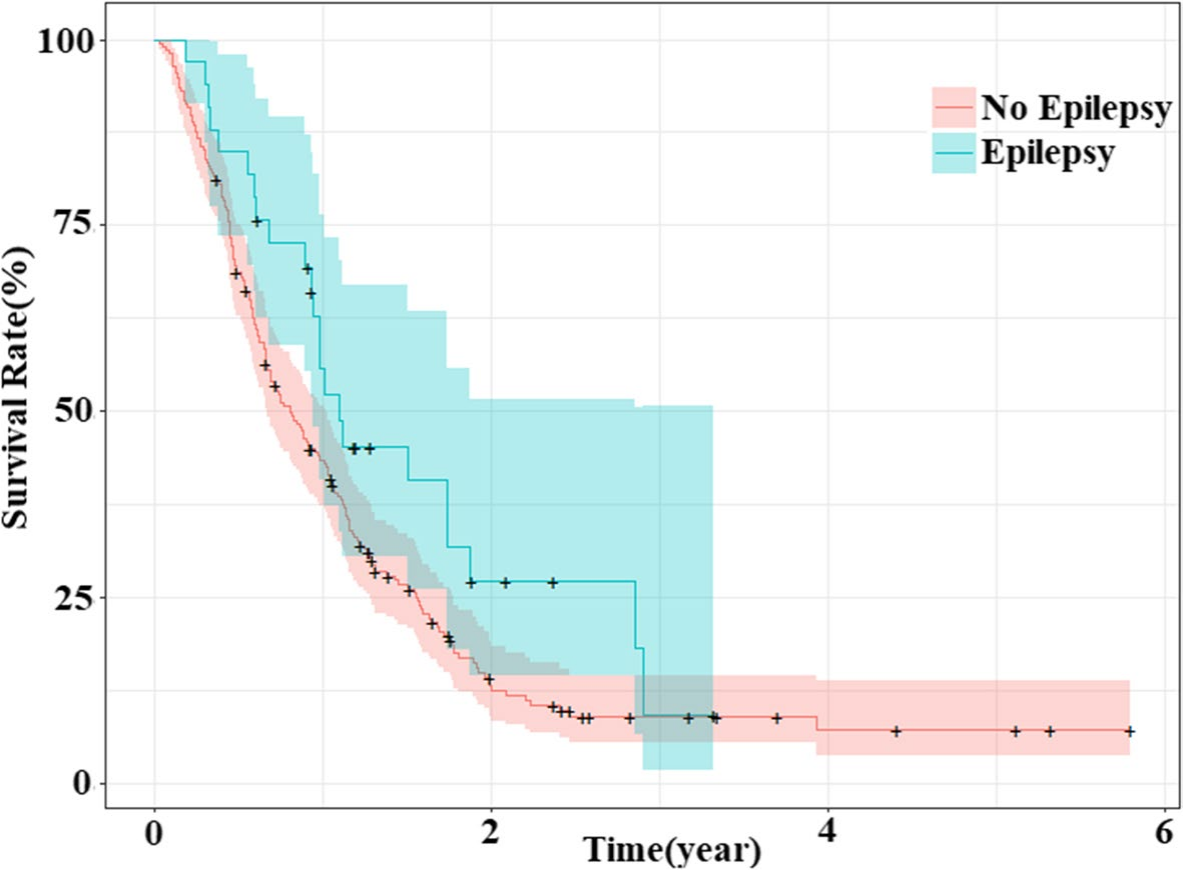
Survival curves of WHO grade IV glioma patients with or without epilepsy. The figure shows survival curves and 95% CI for WHO grade IV glioma patients with and without epilepsy

The effect of epilepsy on survival was insignificant in patients with grades II and III gliomas. Epilepsy was independently associated with improved survival in grade IV glioma patients (Fig. 2). It is noteworthy that although the significance of epilepsy was not consistent between the groups, the 1-year survival rate was higher in the epileptic group than in the non-epileptic group for all tumor grades.

Both chemotherapy and radiation were independently associated with longer survival in grade IV glioma patients. In contrast, radiation and chemotherapy were not associated with survival in grade II tumors.

Lastly, a higher extent of resection (EOR) was associated with prolonged survival for patients with WHO grades II, III, and IV tumors (Table 3). Multiple tumors were a risk factor for survival in patients with grades III and IV tumors. Recurrent tumors also worsened survival in patients with grade IV tumors (Table 3).

**Table 3:** Cox risk regression in WHO grades II, III, and IV gliomas

**Variables**	**Groups**	**WHO grade II**			**WHO grade III**			**WHO grade IV**		
		**Univariate *p*-value**	**Multivariate *p*-value**	**Multivariate HR (95% *CI*)**	**Univariate *p*-value**	**Multivariate *p*-value**	**Multivariate HR (95% *CI*)**	**Univariate *p*-value**	**Multivariate *p*-value**	**Multivariate** **HR (95% *CI*)**
**Epilepsy**	**Yes**	**0.565**	**-**	**-**	**0.362**	**-**	**-**	**0.040**	**0.000**	**0.46 (0.29–0.73)**
	**No**	**Reference**	**-**	**-**	**Reference**	**-**	**-**	**Reference**	**Reference**	**Reference**
**Age**		**0.526**	**-**	**-**	**0.068**	**0.069**	**1.02 (1.00–1.04)**	**0.024**	**0.276**	**1.01 (0.99–1.02)**
**Gender**	**Female**	**Reference**	**-**	**-**	**Reference**	**-**	**-**	**Reference**	**-**	**-**
	**Male**	**0.349**	**-**	**-**	**0.726**	**-**	**-**	**0.169**	**-**	**-**
**Location**	**Supratentorial**	**0.226**	**-**	**-**	**0.005**	**0.001**	**0.27 (0.12–0.60)**	**0.910**	**-**	**-**
	**Subtentorial**	**Reference**	**-**	**-**	**Reference**	**Reference**	**Reference**	**Reference**	**-**	**-**
**Radiotherapy**	**Yes**	**0.547**			**0.009**	**0.005**	**0.40 (0.21–0.76)**	**0.000**	**0.006**	**0.46 (0.27–0.80)**
	**No**	**Reference**	**Reference**	**Reference**	**Reference**	**Reference**	**Reference**	**Reference**	**Reference**	**Reference**
**Chemotherapy**	**Yes**	**0.571**			**0.019**	**0.467**	**1.31 (0.63–2.72)**	**0.000**	**0.038**	**0.56 (0.33–0.97)**
	**No**	**Reference**	**Reference**	**Reference**	**Reference**	**Reference**	**Reference**	**Reference**	**Reference**	**Reference**
**Tumor number**	**Single**	**Reference**	**-**	**-**	**Reference**	**Reference**	**Reference**	**Reference**	**Reference**	**Reference**
	**Multiple**	**0.951**	**-**	**-**	**0.000**	**0.001**	**2.92 (1.10–7.58)**	**0.000**	**0.000**	**2.45 (1.60–3.76)**
**Diagnosis**	**Primary**	**Reference**	**-**	**-**	**Reference**	**-**	**-**	**Reference**	**Reference**	**Reference**
	**Recurrent**	**0.491**	**-**	**-**	**0.513**	**-**	**-**	**0.090**	**0.000**	**2.07 (1.36–3.16)**
**EOR**	**Total**	**Reference**	**-**	**-**	**Reference**	**Reference**	**Reference**	**Reference**	**Reference**	**Reference**
	**Subtotal**	**0.504**	**-**	**-**	**0.686**	**0.160**	**0.61 (0.30–1.22)**	**0.011**	**0.006**	**1.64 (1.15–2.33)**
	**Partial**	**0.003**	**-**	**-**	**0.003**	**0.033**	**2.44 (1.08–5.55)**	**0.003**	**0.014**	**2.44 (1.19–4.98)**
**One-year survival rate**	**Epilepsy**	**87.3%**			**65.8%**			**52.1%**		
	**No epilepsy**	**83.5%**			**55.0%**			**44.3%**		
	**Total**	**86.1%**			**58.9%**			**45.7%**		

## Discussion

This study enrolled 485 glioma patients to report the clinical characteristics of patients with or without glioma-related epilepsy. We also reported the pathological distribution of the gliomas. Based on the information collected at follow-up, we performed a graded Cox risk regression analysis for various variables, including epilepsy. The results showed that epilepsy was associated with improved survival outcomes in GBM patients. Epilepsy is related to age in glioma patients, similar to idiopathic epilepsy [16]. Children and the elderly are more likely to develop idiopathic epilepsy [17]. Contrastingly, our results showed that glioma-related epilepsy was more common in younger people with grades 1 and 2 gliomas. The majority of epilepsy cases occurred in the supratentorial area, and there were only 2 cases of posterior fossa epilepsy. Patients with insular and temporal gliomas were more prone to have epilepsy than gliomas in other lobes. The KPS score is a rating scale used to evaluate the physical condition of cancer patients [18]. No difference in KPS scores was found between the epileptic and non-epileptic groups.

Epilepsy adversely affects cognitive functions, including language, memory, and executive functions [19–21]. Epilepsy is also a significant risk factor for long-term disability [22]. However, epilepsy did not lead to worse performance status for glioma patients at the admission phase in this study. There are certain possible reasons for this discrepancy. For patients, the impact of the tumor on performance status may mask the effects of epilepsy itself. It is also possible that patients with epilepsy are more likely to be admitted to the hospital early, and epilepsy does not significantly affect performance in the short term.

Patients with grade II tumors had the highest epilepsy incidence, followed by those with grades I, III, and IV. Previous studies have also reported that patients with low-grade gliomas had a higher incidence of epilepsy than those with high-grade gliomas [23]. The epileptogenic foci of gliomas are usually located in the cerebral cortex at the outer edge of tumor growth, and there is a small amount of tumor cell infiltration [24]. We hypothesize that epilepsy is more likely to occur when tumors infiltrate the cerebral cortex at an “optimal” level. As epilepsy results from an abnormal cortex discharge, it requires two things: living brain tissue to generate electrical activity and a trigger to switch that discharge from normal to abnormal. Diverse factors can act as triggers, such as immune factors, genetic factors, cerebral trauma, brain tumors, infections, or cerebrovascular diseases [25]. Grade IV gliomas can act as a trigger, but due to their high invasiveness, parts of the brain tissue cannot function, leading to a decrease in the incidence of epilepsy. In grade II tumors, brain tissue is destroyed at an “optimal” level to allow enough “sub-healthy” brain cells to engage in abnormal discharge. As for grades I and grade III tumors, compared to grade II, one has less invasion of brain tissue, and the other has too much invasion of brain tissue. Therefore, they both have lower epilepsy incidence than patients with grade II tumors. In conclusion, patients with grade II tumors had the highest epilepsy incidence, which may have been due to an “optimal” level of infiltration of the cortex. However, the specific mechanism needs to be explored further.

The risk of premature death in people with epilepsy is about two to three times higher than in the general population and up to five times higher in the Asian population [26–28]. Contrastingly, our findings indicated that epilepsy was an independent protective factor for grade IV gliomas. In all three tumor grades studied, the 1-year survival rate in the epileptic group was higher than that in the non-epileptic group. In addition, as we hypothesized above, tumor-related epilepsy requires sufficient “sub-healthy” brain cells. Due to tumor heterogeneity, the invasive ability of tumors of the same grade also varies. Within the same pathological type, epilepsy may indicate that the cancer is less aggressive as it provides more “sub-healthy” brain cells rather than directly killing them. As reported earlier, we found a higher occurrence of glioma-associated epilepsy in younger people. The better physical condition of young people compared to the elderly may be one of the reasons for the protective effect of epilepsy. The role of molecular pathology in the diagnosis of glioma is increasing compared to the classification methods in 2007, 2016, and 2021, and our vision is no longer limited to the microscope [29–32]. The study of glioma-associated epilepsy may bring a new perspective to molecular pathology-based diagnostic approaches.

Both glioma and epilepsy are recognized as intractable diseases. Glutamate plays an essential role in epilepsy pathogenesis, and its concentration is more than 10 times higher around gliomas than in normal brain tissues [13]. The mechanisms for this accumulation are as follows: (1) glioma cells overexpress the cystine-glutamate transporter, which increases extracellular glutamate concentrations [11, 12, 33], (2) decreased expression of EAAT1 and EAAT2 (two excitatory amino acid transporters) in tumor cell membranes and peritumoral activated microglia lead to reduced glutamate reuptake [12, 33], and (3) although IDH mutations do not directly produce glutamate, they can lead to the production of D-2-hydroxyglutarate, which stimulates glutamate receptors due to its structural similarity to glutamate [34, 35].

However, glutamate and IDH mutations have entirely different consequences. The overexpression of the cystine-glutamate transporter brings glutamate to the outside of the cell and cystine into the cell. Cystine is the raw material for glutathione synthesis, which helps tumor cells resist free radical attacks and promotes tumor growth [36]. IDH mutations impede cellular metabolism and hinder tumor growth in three main ways: (1) under hypoxic conditions, mutant IDH1 cannot undergo reductive carboxylation, (2) the chemical reaction induced by mutant IDH consumes NADPH, so tumors are more susceptible to free radical damage, (3) D-2-hydroxyglutarate directly inhibits ATP synthase by binding to it and affects ATP production under limiting glucose conditions [37].

Both glutamate and IDH mutations can trigger epilepsy. However, high glutamate concentrations can imbalance the glutamate/glutamine cycle between astrocytes and neurons, resulting in the swelling of astrocytes and neuronal death [37]. In this case, epilepsy onset may be limited. The incidence of glioma-associated epilepsy is highly correlated with IDH mutations [38], and IDH mutations tend to be associated with prolonged survival [39]. This suggests that IDH mutations play a more important role than glutamate in epilepsy pathogenesis in gliomas. It also explains, to a certain extent, why the survival rate of patients in the epilepsy group was higher than that in the non-epilepsy group.

We would like to emphasize that while epilepsy was associated with better survival in patients with glioma, it does not mean that epilepsy is good for the patients. In fact, epilepsy has been shown to promote the proliferation of glioma cells [14]. Epilepsy also damages or kills neurons, making room for tumor growth [11, 33]. Taken together, it is crucial to consider the co-treatment of epilepsy and tumors.

In our study, radiotherapy and chemotherapy were not associated with survival in grade II glioma patients. This could be because not all grade II patients require routine radiotherapy and chemotherapy according to the glioma treatment guidelines (whereas all grade IV patients require postoperative radiotherapy and chemotherapy). Among grade II tumor patients, some patients with good genotypes will not require radiotherapy and chemotherapy. However, some patients who need radiotherapy and chemotherapy post-surgery may be unable to get the treatment due to their will or their families’ will or economic reasons.

Next, we highlight certain limitations of this study. Even though we recruited a large number of patients, the results of this study were based on a current single-center dataset and are not representative of all glioma patients. In the future, it is necessary to establish a multicenter database of glioma patients to improve the universality of the conclusions obtained. Next, we studied glioma-related epilepsy from a macro perspective, so further studies on the individual level need to be conducted. Finally, we discussed the reasons for the “protection” afforded by epilepsy, and the possible reasons, which need to be further verified.

## Conclusion

Our study showed that younger patients and patients with grade II tumors were most likely to have glioma-related epilepsy, whereas older people and patients with grade IV tumors were least likely. Epilepsy did not lead to worse admission performance in glioma patients. Lastly, GBM patients had the lowest survival rates compared to patients with other glioma types, but epilepsy increased the survival rate.

## Supplementary Information

**Additional file 1: Supplementary Table 1.** General characteristics of grade I glioma. **Supplementary Table 2.** General characteristics of grade II glioma. **Supplementary Table 3.** General characteristics of grade III glioma. **Supplementary Table 4.** General characteristics of grade IV glioma. **Supplementary Table 5.** Location difference between high-grade glioma and low-grade glioma.

## Data Availability

The datasets used and analyzed during the current study are available from the corresponding author on reasonable request.

## Abbreviations

LGG: Low-grade glioma
GBM: Glioblastoma
KPS: Karnofsky Performance Status
EEG: Electroencephalogram
SD: Standard deviation
KM: Kaplan-Meier
OR: Odds ratios
HR: Hazard ratios
CI: Confidence intervals
IDH: Isocitrate dehydrogenase
EOR: Extent of resection
EAAT: Excitatory amino acid transporters
