# Supplementary material for: Does epilepsy always indicate worse outcomes? A longitudinal follow-up analysis of 485 glioma patients
Source: World J Surg Oncol. 2022 Sep 19;20:297. doi: 10.1186/s12957-022-02772-2 (PMC9484070; doi:10.1186/s12957-022-02772-2)
Supplement: Supplementary file 1 — Additional file 1: Supplementary Table 1. General characteristics of grade I glioma. Supplementary Table 2. General characteristics of grade II glioma. Supplementary Table 3. General characteristics of grade III glioma. Supplementary Table 4. General characteristics of grade IV glioma. Supplementary Table 5. Location difference between high-grade glioma and low-grade glioma. [file 12957_2022_2772_MOESM1_ESM.docx]

| Supplementary Table 1: General characteristics of grade I glioma | | | | | | | |
| --- | --- | --- | --- | --- | --- | --- | --- |
| Variables | Groups | Epilepsy | No Epilepsy | Total | Univariate *p* value | Multivariate *p* value | Multivariate  OR (95%CI) |
| Gender | Female | 8(20.00%) | 13(32.50%) | 21(52.50%) | 0.251 | NA |  |
|  | Male | 4(10.00%) | 15(37.50%) | 19(47.50%) | Reference |  |  |
| Age(years) | | 24.92±9.29 | 37.79±18.48 | 33.93±17.31 | 0.031 | 0.022 | 0.99(0.98-1.00) |
| Location | Frontal lobe | 2(5.00%) | 3(7.50%) | 5(12.50%) | 0.204 | 0.237 | 1.27(0.86-1.86) |
|  | Temporal lobe | 5(12.50%) | 2(5.00%) | 7(17.50%) | 0.003 | 0.002 | 1.80(1.29-2.52) |
|  | Parietal lobe | 2(5.00%) | 2(5.00%) | 4(10.00%) | 0.115 | 0.131 | 1.40(0.92-2.13) |
|  | Occipital lobe | 0(0.00%) | 1(2.50%) | 1(2.50%) | 0.764 | 0.804 | 0.90(0.98-2.00) |
|  | Insula | 0(0.00%) | 0(0.00%) | 0(0.00%) | NA | NA | NA |
|  | Others | 3(7.50%) | 20(50.00%) | 23(57.50%) | Reference |  |  |
| Tumor number | Single | 12(30.00%) | 28(70.00%) | 40(100.00%) | NA | NA |  |
|  | Multiple | 0(0.00%) | 0(0.00%) | 0(0.00%) | Reference |  |  |
| Admission status | KPS ≤ 70 | 0(0.00%) | 0(0.00%) | 0(0.00%) | NA | NA |  |
|  | KPS > 70 | 12(30.00%) | 28(70.00%) | 40(100.00%) | Reference |  |  |
| Type | Primary | 12(30.00%) | 26(65.00%) | 38(95.00%) | 0.355 | NA |  |
|  | Recurrent | 0(0.00%) | 2(5.00%) | 2(5.00%) | Reference |  |  |
| Total |  | 12(30.00%) | 28(70.00%) | 40(100.00%) |  |  |  |

| Supplementary Table 2: General characteristics of grade II glioma | | | | | | | |
| --- | --- | --- | --- | --- | --- | --- | --- |
| Variables | Groups | Epilepsy | No Epilepsy | Total | Univariate *p* value | Multivariate *p* value | Multivariate  OR (95%CI) |
| Gender | Female | 30(27.78%) | 34(31.48%) | 64(59.26%) | 0.314 | NA | NA |
|  | Male | 25(23.15%) | 19(17.59%) | 44(40.74%) | Reference |  |  |
| Age(years) | | 39.36±16.05 | 47.75±15.40 | 43.48±16.28 | 0.007 | 0.000 | 0.99(0.98-1.00) |
| Location | Frontal lobe | 25(23.15%) | 24(22.22%) | 49(45.37%) | 0.024 | 0.006 | 1.60(1.16-2.23) |
|  | Temporal lobe | 18(16.67%) | 7(6.48%) | 25(23.15%) | 0.002 | 0.000 | 1.89(1.33-2.68) |
|  | Parietal lobe | 3(2.78%) | 6(5.56%) | 9(8.33%) | 0.329 | 0.075 | 1.49(0.97-2.30) |
|  | Occipital lobe | 0(0.00%) | 2(1.85%) | 2(1.85%) | 0.768 | 0.417 | 0.74(0.37-1.51) |
|  | Insula | 8(7.41%) | 6(5.56%) | 14(12.96%) | 0.027 | 0.003 | 1.84(1.24-2.72) |
|  | Others | 1(0.93%) | 8(7.41%) | 9(8.33%) | Reference |  |  |
| Tumor number | Single | 53(49.07%) | 52(48.15%) | 105(97.22%) | 0.584 | NA | NA |
|  | Multiple | 2(1.85%) | 1(0.93%) | 3(2.78%) | Reference |  |  |
| Admission status | KPS ≤ 70 | 2(1.85%) | 2(1.85%) | 4(3.70%) | 0.97 | NA | NA |
|  | KPS > 70 | 53(49.07%) | 51(47.22%) | 104(96.30%) | Reference |  |  |
| Type | Primary | 53(49.07%) | 48(44.44%) | 101(93.52%) | 0.225 | NA | NA |
|  | Recurrent | 2(1.85%) | 5(4.63%) | 7(6.48%) | Reference |  |  |
| Total |  | 55(50.93%) | 53(49.07%) | 108(100.00%) |  |  |  |

| Supplementary Table 3: General characteristics of grade III glioma | | | | | | | |
| --- | --- | --- | --- | --- | --- | --- | --- |
| Variables | Groups | Epilepsy | No Epilepsy | Total | Univariate *p* value | Multivariate *p* value | Multivariate  OR (95%CI) |
| Gender | Female | 5(5.75%) | 35(40.23%) | 40(45.98%) | 0.053 | 0.020 | 1.23(1.04-1.47) |
|  | Male | 14(16.09%) | 33(37.93%) | 47(54.02%) | Reference |  |  |
| Age(years) | | 46.95±11.27 | 52.53±16.37 | 51.39±15.58 | 0.163 | NA | NA |
| Location | Frontal lobe | 12(13.79%) | 30(34.48%) | 42(48.28%) | 0.019 | 0.015 | 1.33(1.06-1.68) |
|  | Temporal lobe | 4(4.60%) | 9(10.34%) | 13(14.94%) | 0.046 | 0.023 | 1.41(1.06-1.89) |
|  | Parietal lobe | 1(1.15%) | 7(8.05%) | 8(9.20%) | 0.480 | 0.732 | 1.06(0.76-1.49) |
|  | Occipital lobe | 0(0.00%) | 3(3.45%) | 3(3.45%) | 1.000 | 0.848 | 1.05(0.64-1.71) |
|  | Insula | 2(2.30%) | 3(3.45%) | 5(5.75%) | 0.059 | 0.036 | 1.54(1.04-2.30) |
|  | Others | 0(0.00%) | 16(18.39%) | 16(18.39%) | Reference |  |  |
| Tumor number | Single | 18(20.69%) | 60(68.97%) | 78(89.66%) | 0.417 | NA | NA |
|  | Multiple | 1(1.15%) | 8(9.20%) | 9(10.34%) | Reference |  |  |
| Admission status | KPS ≤ 70 | 4(4.60%) | 13(14.94%) | 17(19.54%) | 0.853 | NA | NA |
|  | KPS > 70 | 15(17.24%) | 55(63.22%) | 70(80.46%) | Reference |  |  |
| Type | Primary | 14(16.09%) | 55(63.22%) | 69(79.31%) | 0.499 | NA | NA |
|  | Recurrent | 5(5.75%) | 13(14.94%) | 18(20.69%) | Reference |  |  |
| Total |  | 19(21.84%) | 68(78.16%) | 87(100.00%) |  |  |  |

| Supplementary Table 4: General characteristics of grade IV glioma | | | | | | | |
| --- | --- | --- | --- | --- | --- | --- | --- |
| Variables | Groups | Epilepsy | No Epilepsy | Total | Univariate *p* value | Multivariate *p* value | Multivariate  OR (95%CI) |
| Gender | Female | 16(6.40%) | 99(39.60%) | 115(46.00%) | 0.76 | NA | NA |
|  | Male | 17(6.80%) | 118(47.20%) | 135(54.00%) | Reference |  |  |
| Age(years) | | 56.82±12.97 | 58.53±11.53 | 58.30±11.74 | 0.439 | NA | NA |
| Location | Frontal lobe | 15(6.00%) | 74(29.60%) | 89(35.60%) | 0.285 | NA | NA |
|  | Temporal lobe | 8(3.20%) | 77(30.80%) | 85(34.00%) | 0.774 | NA | NA |
|  | Parietal lobe | 4(1.60%) | 23(9.20%) | 27(10.80%) | 0.458 | NA | NA |
|  | Occipital lobe | 4(1.60%) | 20(8.00%) | 24(9.60%) | 0.373 | NA | NA |
|  | Insula | 1(0.40%) | 9(3.60%) | 10(4.00%) | 0.811 | NA | NA |
|  | Others | 1(0.40%) | 14(5.60%) | 15(6.00%) | Reference |  |  |
| Tumor number | Single | 27(10.80%) | 191(76.40%) | 218(87.20%) | 0.323 | NA | NA |
|  | Multiple | 6(2.40%) | 26(10.40%) | 32(12.80%) | Reference |  |  |
| Admission status | KPS ≤ 70 | 9(3.60%) | 55(22.00%) | 64(25.60%) | 0.814 | NA | NA |
|  | KPS > 70 | 24(9.60%) | 162(64.80%) | 186(74.40%) | Reference |  |  |
| Type | Primary | 27(10.80%) | 191(76.40%) | 218(87.20%) | 0.323 | NA | NA |
|  | Recurrent | 6(2.40%) | 26(10.40%) | 32(12.80%) | Reference |  |  |
| Total |  | 33(13.20%) | 217(86.80%) | 250(100.00%) |  |  |  |

| Supplementary Table 5: Location difference between high-grade glioma and low-grade glioma. | | |
| --- | --- | --- |
| Location | p value | OR (95%CI) |
| Frontal lobe | 0.001 | 1.241(1.091-1.412) |
| Temporal lobe | 0.000 | 1.299(1.134-1.488) |
| Parietal lobe | 0.006 | 1.268(1.070-1.502) |
| Occipital lobe | 0.000 | 1.504(1.236-1.830) |
| Insula | 0.804 | 1.025(0.841-1.251) |
| Others | Reference | |
